# Prevalence and Risk Factors of Deep Vein Thrombosis in Patients Undergoing Lumbar Interbody Fusion Surgery: A Single-Center Cross-Sectional Study

**DOI:** 10.1097/MD.0000000000002205

**Published:** 2015-12-07

**Authors:** Si-Dong Yang, Wen-Yuan Ding, Da-Long Yang, Yong Shen, Ying-Ze Zhang, Shi-Qing Feng, Feng-Dong Zhao

**Affiliations:** From the Department of Spinal Surgery, The Third Hospital of Hebei Medical University, 139 Ziqiang Road (S-DY, W-YD, D-LY, YS); Hebei Provincial Key Laboratory of Orthopedic Biomechanics, 139 Ziqiang Road, Shijiazhuang (W-YD, D-LY, YS, Y-ZZ); Department of Orthopedic Surgery, Tianjin Medical University General Hospital, 154 Anshan Road, Tianjin (S-QF); and Department of Orthopedic Surgery, Sir Run Run Shaw Hospital, Zhejiang University School of Medicine, 3 East Qingchun Road, Hangzhou, PR China (F-DZ).

## Abstract

This cross-sectional study was designed to obtain the current prevalence of deep vein thrombosis (DVT) and analyze related risk factors in patients undergoing lumbar interbody fusion.

Medical record data were collected from Department of Spinal Surgery, The Third Hospital of Hebei Medical University, between July 2014 and March 2015. Both univariate analysis and binary logistic regression analysis were performed to determine risk factors for DVT.

A total of 995 patients were admitted into this study, including 484 men and 511 women, aged from 14 to 89 years old (median 50, IQR 19). The detection rate of lower limb DVT by ultrasonography was 22.4% (223/995) in patients undergoing lumbar interbody fusion. Notably, average VAS (visual analog scale) score in the first 3 days after surgery in the DVT group was more than that in the non-DVT group (*Z* = −21.69, *P* < 0.001). The logistic regression model was established as logit *P* = −13.257 + 0.056∗X_1_ − 0.243∗X_8_ + 2.085∗X_10_ + 0.001∗X_12_, (X_1_ = age; X_8_ = HDL; X_10_ = VAS; X_12_ = blood transfusion; *x*^2^ = 677.763, *P* < 0.001).

In conclusion, advanced age, high postoperative VAS scores, and blood transfusion were risk factors for postoperative lower limb DVT. As well, the logistic regression model may contribute to an early evaluation postoperatively to ascertain the risk of lower limb DVT in patients undergoing lumbar interbody fusion surgery.

## INTRODUCTION

Venous thromboembolism (VTE) consists of 2 interrelated conditions caused by blood clots, notably deep vein thrombosis (DVT) and pulmonary embolism (PE).^1^ Deep vein thrombosis is often overlooked, but a serious, and potentially preventable disorder that affects mostly hospitalized patients due to immobility. Deep vein thrombosis is composed of a clot of blood formed in sites of damaged vessels and areas of stagnant blood flow such as lower leg, thigh, or pelvis. This clot may remain in situ or move to the pulmonary arteries and cause PE. It can lead to severe morbidity with poor quality of life and even sudden death related to PE. Approximately half of all untreated DVT cases are complicated by PE, and conversely, 50% to 80% of all untreated PE cases are associated with DVT.^2,3^ The risk factors that have the strong odds for a potential VTE include, but not limited to, major general surgery, major orthopedic surgery, spinal cord injury, fracture of the pelvis, hip or long bones; multiple trauma, malignancy, congestive heart or respiratory failure, myocardial infarction, prior VTE, advancement in age, obesity, immobility, varicose veins, pregnancy and puerperium, use of oral contraceptives, antiphospholipid antibody syndrome, and such hereditary risk factors as antithrombin and protein C and S deficiencies.^4–7^ In spinal surgery, the factors for venous stasis are considered to be long-time horizontal ventral decubitus, lack of muscle tone, and venous compression by retractors and postoperative bed rest.^8^

It has been reported that venous intimal injury may occur in surgical handling.^8^ Consistent with this, our recent cohort study has revealed that shorter incision length during operation is more likely to contribute to lower limb DVT.^9^ In the same study, we have found that the patients undergoing spinal interbody fusion are the main population especially vulnerable to lower limb DVT.^9^ It is estimated that the DVT incidence of asymptomatic patients exceeds 15%.^10^ Thus, it is very necessary to detect lower limb DVT by routine use of ultrasonography to the patients undergoing spinal interbody fusion, because it may lead to potentially lethal disease such as PE, although DVT incidence of symptomatic patients is only around 0.5% to 2.5% of the procedures in the spine.^10^ In addition, we have noticed in clinical situations that the patients after surgery tend to do less lower limb exercise due to lower back pain. Therefore, visual analog scale (VAS) score is analyzed as a risk factor in this study to explore its relation to postoperative lower limb DVT.

Based on the above-mentioned reasons, this single-center cross-sectional study is designed with the purpose to investigate the current prevalence of lower limb DVT in the patients undergoing spinal interbody fusion surgery through the detection rate of lower limb DVT by ultrasonography, and in the meanwhile, to explore the risk factors associated with lower limb DVT. This study is expected to lead to a better understanding of the lower limb DVT after spine interbody fusion surgery, thus contributing to an early primary evaluation of postoperative lower limb DVT risk rate.

## PATIENTS AND METHODS

### Ethics Statement

There is no need to seek consent from patients, since in this retrospective cross-sectional study all the data were collected and analyzed anonymously without any potential harm to the patients; this is approved by Ethics Committee of The Third Hospital of Hebei Medical University.

### Patients

This study included patients who underwent lumbar interbody fusion operations, admitted into Department of Spinal Surgery, The Third Hospital of Hebei Medical University in China, between July 2014 and March 2015. The inclusion criteria were complete medical records including patient number, sex, age, body weight, body height, regional distribution, hospital stay, occupation, DVT, spinal epidural hematoma, hypertension, diabetes, heart disease, surgical method, level and number of vertebrae fusion, operation duration, blood loss, blood transfusion, incision length, prothrombin time activity (PTA), fibrinogen (FIB), thrombin time (TT), D-dimer, HDL (high-density lipoprotein), LDL (low-density lipoprotein), total cholesterol (TC), total bilirubin, direct bilirubin and indirect bilirubin, as well as VAS score in the first 3 postoperative days. All of the above biochemical factors were tested before spine surgery. All the patients with lumbar interbody fusion surgery were examined by lower extremity ultrasonography pre- and postoperatively. After spine surgery, subfascial drainage was routinely used. Besides, all the patients routinely received prophylactic treatment with low-molecular-weight heparin (LMWH) at 4100 IU per day. Simultaneously, the patients were encouraged to do lower limb exercise on bed to accelerate blood circulation. The exclusion criteria included patients who did not undergo lumbar interbody fusion, had a prior DVT, or had been on anticoagulant therapy such as warfarin, aspirin, and clopidogrel prior to hospitalization.

## METHODS

Data was collected from subjects after they were identified according to the inclusion and exclusion criteria. Microsoft Excel was used for the data input. Statistical analyses were performed using SPSS for Windows, version 18.0 (SPSS, Inc, Chicago, IL). All measurement data were presented as the median value (interquartile range, IQR). Chi square and Mann–Whitney *U* test were used for data analyses where applicable. The nonconditional binary logistic regression model was used for the exploration of the associated risk factors of lower limb DVT in postspinal surgery patients. Values for *P* < 0.05 were regarded as significant with 2-tailed tests.

## RESULTS

### General Data of Patients Included

A total of 995 patients, including 484 men and 511 women, met the criteria and were therefore incorporated into this single-center cross-sectional study. Their ages ranged from 14 to 89 years (median 50, IQR 19), and demographically, 668 patients hailed from rural areas and 327 from urban areas. The average hospital stay was 15 days (IQR, 4). Totally 223 cases (22.4%) developed postoperative DVT, which were all anonymous. One DVT site was found with 152 DVT cases, 2 DVT sites with 60 cases, and 3 DVT sites with 11 cases. With regard to DVT distribution, 208 sites were found in venous plexus of calf muscle, 52 DVT sites in posterior tibial veins, 30 in popliteal vein, 7 in calf superficial vein, and only 1 in femoral vein. In addition, no PE cases were found, and only 1 case (0.1%) developed spinal epidural hematoma.

### Age, Gender, and Regional Distribution

The average age of the DVT group was 57 years (IQR = 13) whereas that of the non-DVT group was 48 years (IQR = 23). Mann–Whitney *U* test showed a statistically significant difference between the age of DVT patients and non-DVT group (*Z* = −8.692, *P* < 0.001). Out of the 233 postoperative DVT patients, 103 were men and 120 were women, with 166 and 57 of the cases from rural and urban areas, respectively, whereas 381 men and 391 women were also identified in the 772 non-DVT cases with 502 and 270 cases from rural and urban areas respectively. The chi square test indicated that there was no statistically significant difference in gender distribution between DVT group and non-DVT group (*x*^2^ = 0.693, *P* = 0.405), but regional distribution was significantly different (*x*^2^ = 6.949, *P* = 0.008).

### Comparison of Surgical Data

The surgical data included surgical duration, blood loss, blood transfusion, and incision length according to the patient operation note. Table 1 shows that blood loss and blood transfusion in the DVT group was significantly more than that in the non-DVT group (Mann–Whitney *U* test, *P* = 0.000 and *P* = 0.001, respectively). Also, incision length in the DVT group was significantly shorter than that in the non-DVT group (Mann–Whitney *U* test, *P* = 0.037). However, surgical duration between the 2 groups did not show any statistical difference (Mann–Whitney *U* test, *P* = 0.108).

**TABLE 1:**
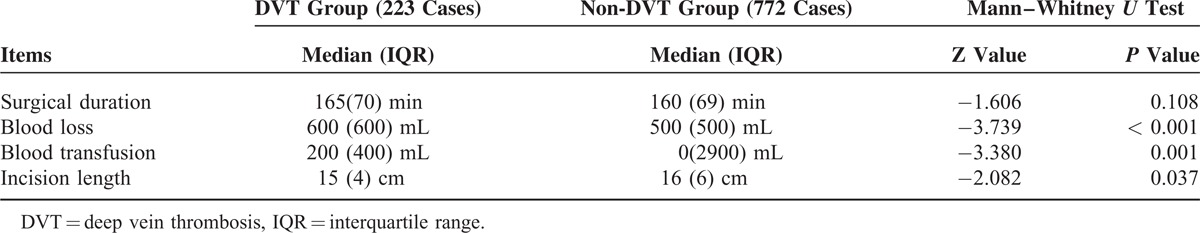
Comparison of Surgical Data Associated With Postoperative DVT

### Chronic Disease History

Table 2 shows comparison of chronic disease history between the DVT group and non-DVT group, including high blood pressure (HBP), diabetes mellitus (DM), and heart disease (HD). High blood pressure in the DVT group was more than that in the non-DVT group (chi square test, *P* < 0.001), whereas DM and HD between the 2 groups did not show any statistically significant difference.

**TABLE 2:**
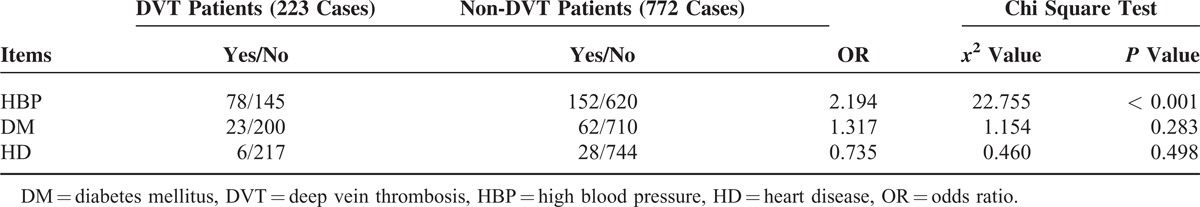
Comparison of Chronic Disease History Associated With Postoperative DVT

### Biochemical Analyses

Table 3 shows the biochemical analyses associated with postoperative DVT conducted between the DVT group and the non-DVT group. There was a statistically significant difference between the 2 groups in FIB, HDL, LDL, and TC (Mann–Whitney *U* test, *P* = 0.025, *P* = 0.013, *P* = 0.006, and *P* = 0.002, respectively), whereas there was no statistical difference among the other analytes including PTA and TT (*P* = 0.163 and *P* = 0.518, respectively; Mann–Whitney *U* test).

**TABLE 3:**
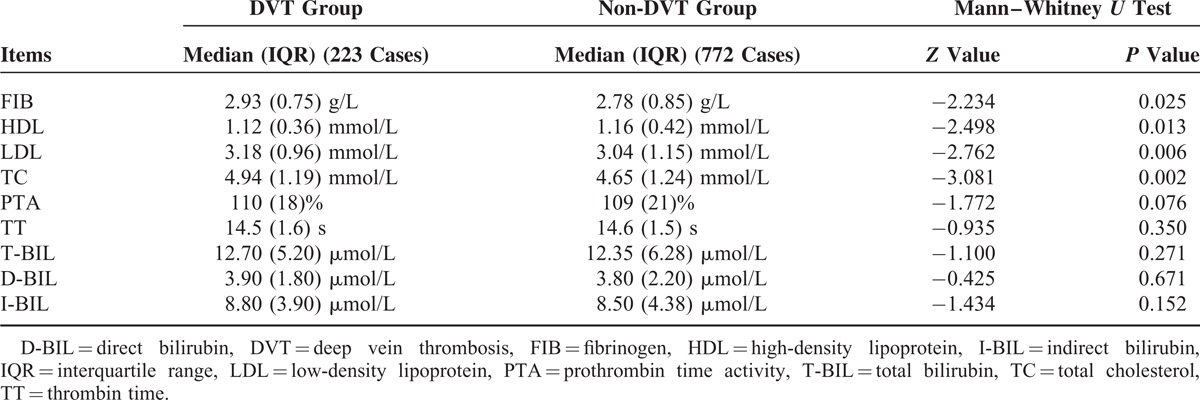
Biochemical Analyses Associated With Postoperative DVT

### DVT and D-Dimer

The cutoff D-dimer used in this study is 0.3 mg/L. In the DVT group (n = 223), there are 179 cases with D-dimer ≤ 0.3 mg/L and 44 cases with D-dimer > 0.3 mg/L. Meanwhile, in the non-DVT group (n = 772), there are 688 cases with D-dimer ≤ 0.3 mg/L and 84 cases with D-dimer > 0.3 mg/L. It was found that the proportion of patients with D-dimer > 0.3 mg/L in the DVT group was bigger than that in the non-DVT group (Pearson chi square test, χ^2^ = 12.09, *P* = 0.001). And then, we compared the data with D-dimer > 0.3 mg/L between the DVT group (n = 44) and non-DVT group (n = 84). The results showed that the level of D-dimer was 0.495 (IQR = 0.52) mg/L in the DVT group and 0.490 (IQR = 0.43) mg/L in the non-DVT group, without statistical significance (Mann–Whitney *U* test, *Z* = −1.124, *P* = 0.261).

### Hospital Stay and BMI

The average hospital stay in the DVT group was 16(IQR = 5) days and in the non-DVT group was 15(IQR = 4) days. The difference between them was statistically significant (Mann–Whitney *U* test, *Z* = −4.339, *P* < 0.001). In addition, the body mass index (BMI) was calculated according to body height and weight in all 995 cases, including 223 cases in the DVT group and 772 cases in the non-DVT group. Body mass index in the DVT group was 25.81(IQR = 4.15) and in the non-DVT group 25.39 (IQR = 4.00), without significant difference (Mann–Whitney *U* test, *Z* = −1.412, *P* = 0.158).

### DVT and Interbody Fusion Level

In this study, all 995 patients underwent different levels of interbody fusion. A total of 731 cases underwent single-level interbody fusion including 156 DVT cases, 218 cases underwent double-level interbody fusion including 51 DVT cases, and 46 cases underwent three-level and above of interbody fusion including 16 DVT cases. The chi square test applied to the analyses of this data showed no significant difference in DVT incidence in relation to fusion levels (χ^2^ = 4.652, *P* = 0.098).

### DVT and VAS Score

Average VAS score in the first 3 days after surgery was 3.33 (IQR = 0.67) in the non-DVT group, and 6.00 (IQR = 1.67) in the DVT group. The difference was statistically significant between the 2 groups (Mann–Whitney *U* test, *Z* = −21.69, *P* < 0.001).

### Logistic Regression Analysis

All patients were grouped into 4 categories for the purpose of analysis according to their age (∼45; 45–54; 55–64; 65). The regression analysis method was set to be Backward (LR) and probability for stepwise (Entry 0.10, Removal 0.15). As shown in Table 4, the binary logistic regression revealed advanced age, high VAS scores, and large amount of blood transfusion as the risk factors for postoperative DVT. High-density lipoprotein was however a likely protective factor for postoperative DVT. Hence, the logistic regression equation was presented as logit *P* = −13.257+ 0.056∗X_1_− 0.243∗X_8_+ 2.085∗X_10_ + 0.001∗X_12_, (X_1_ = age; X_8_ = HDL; X_10_ = VAS; X_12_ = blood transfusion). The regression model established in this study was statistically significant by the chi square test (χ^2^ = 677.763, *P* < 0.001).

**TABLE 4:**
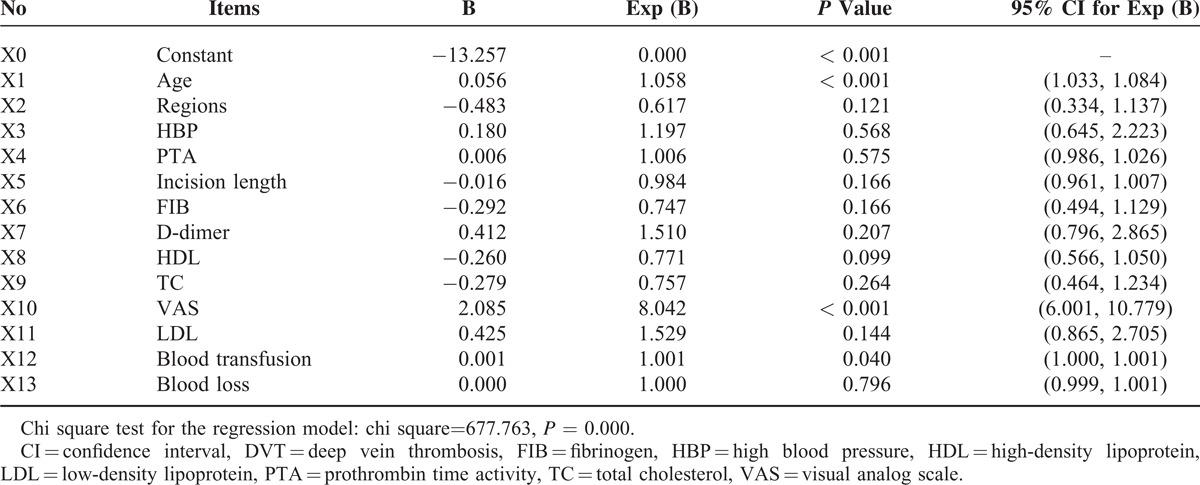
Binary Logistic Regression Analysis of Postoperative DVT

## DISCUSSION

In this study, it was found by univariate analysis that advanced age, blood loss, blood transfusion, incision length, regional distribution, hypertension, hospital stay, VAS score following surgery, FIB, HDL, LDL, and TC, were all significantly different between the patients with and without DVT. However, the regression model indicated that only advanced age, high VAS scores during the first 3 days after surgery, and large amount of blood transfusion were risk factors for lower limb DVT in patients undergoing spinal interbody fusion. On the contrary, HDL was more likely a protective factor, not a risk factor for postoperative DVT incidence.

To our knowledge, it has been widely reported that postoperative D-dimer assay can effectively predict DVT occurrence.^7,11–16^ However, even though high D-dimer values are of significance in the sensitivity and specificity as a risk factor for DVT, it may not be accurate to make a diagnosis, since the choice of the cutoff values usually depends on the methods used and the populations studied. In terms of the present study, only preoperative D-dimer was obtained. We have compared positive and negative DVT to positive and negative D-dimers. Taken all together, although the proportion of patients with D-dimer > 0.3 mg/L in the DVT group was bigger than that in the non-DVT group, the distributional trend of D-dimer in patients with D-dimer > 0.3 mg/L is the same between the DVT group and the non-DVT group. Thus, we cannot draw an exact conclusion from the results regarding D-dimer.

This study confirmed the anticipated advanced age as a risk factor for postoperative DVT after spinal interbody fusion, as reported in other studies as well.^4,18–20^ The result of this study indicated a total DVT incidence among patients with the mean age of 50 years (IQR = 19) as 22.4% after spinal surgery.^4^ The outcome of this study however, seemed inconsistent with a similar research by Strom et al, involving patients with significantly higher age group (mean 64), that reported only 4.3% VTE incidence (including acute/chronic DVT and PE) after spinal surgery, because lower extremity ultrasonography was performed only on patients that showed symptoms of DVT (unilateral calf pain, edema, erythema, warmth) as well as those patients who remained immobilized on the 3rd postoperative day.^4^ On the other hand, total DVT incidence was higher (22.4%) in this study because lower extremity ultrasonography was routinely performed on all the patients on the 7th postoperative day. The patients were routinely advised by the doctors to walk after the 7th postoperative day. Lower extremity ultrasonography was thus used to rule out the DVT-associated complications. However, DVT incidence has been reported to be ∼0.5% to 2.5% for symptomatic patients and 15% to 31% for asymptomatic patients after the spinal surgery procedures, which is in line with the 22.4% DVT incidence found in this study.^10,21^

Lumbar spine has been found to be more prone to defects than the other regions of the whole spine.^22^ Research indicates that ∼15% of patients undergoing posterior spinal surgery develop DVT if prophylactic measures are not taken, implying a need to make prophylactic measures part of spinal surgery procedures.^23^ Making prophylaxis part of spinal surgery procedures is vital for VTE prevention, which is more economical than treatment. Although prophylactic treatment has proven effective in preventing hospitalized patients from VTE, only one-third of them receive adequate prophylactic treatment.^24^ The 2 commonly identified prophylactic measures are mechanical prophylaxis involving intermittent pneumatic compression, and pharmacological prophylaxis, mainly including LMWH.^25^ However, the effect of LMWH prophylaxis has received bitter controversies in some studies.^4,26–28^

Pathological and surgical sites or surgical procedure in orthopedics has identified hip or knee arthroplasty, lower limb fracture, and spinal cord injury as high risk for VTE incidence, contrary to upper limb surgery that has comparatively lower risk.^29–31^ Although there have been several studies about DVT after spinal surgery, the exact incidence of PE and DVT after spinal interbody fusion has not been established. This is confirmed by the result of several studies conducted to ascertain DVT incidence in single center, same prophylaxis, and routine screening for DVT, which had VTE incidence ranging from 1% to 25.0% due to differences in the surgical procedure and surgical site.^10,11,19,20,23,32^ The radiological study conducted by Takahashi et al to evaluate 100 patients using contrast-enhanced CT after spine surgery reports asymptomatic PE and DVT in the patients as 18% and 19% respectively.^17^ A multi-institutional study by Hohl et al about patients who underwent spinal surgery, also reported 1.5%, 0.88%, and 0.66% as the prevalence rate for VTE, symptomatic PE and DVT respectively.^33^ Epidural hematoma is a serious complication after spinal surgery that results in spinal paresis. The result of this study showed epidural hematoma incidence of 0.1% which is consistent with that of the symptomatic postoperative epidural hematoma incidence ranging from 0.1% to 3.0% being reported.^34,35^

A recent prospective clinical study reported that female sex, advanced age, spinal level, and neurological deficits, were all risk factors for postoperative DVT after elective spinal surgery.^20^ Cervical spinal surgery in particular has an associated low risk of only 3.0%, and in patients with PE, 3 out of 4 have no DVT, indicating that screening for PE is also needed in high-risk patients since DVT is not the only cause of PE in cervical spinal surgery.^20^ It is an undisputable fact that PE is more critical and potentially fatal than DVT. However, since PE is rare, many researchers assume it is preceded by DVT hence their focus on DVT. Even thrombi of the calf veins have the potential of proximal propagation, ultimately resulting in PE.^20^ Patients with total joint arthroplasty of the lower extremities have an increased rate of fatal PE due to asymptomatic DVT.^36^ This therefore justifies the routine screening of patients undergoing spinal surgery for DVT.

Previous studies reported that major surgery had a high risk of VTE.^32,33,37^ Multilevel spinal instrumented fusion surgery also induces long operative time, prolonged bed rest, much bleeding, as well as much blood transfusion. These factors result in a high risk of VTE associated with decompression surgery and posterior lumbar interbody fusion. It is in line with the findings reported in the present study that blood transfusion is a risk factor for the postoperative DVT.

In this study, it is of note that the high VAS score following surgery was found for the first time as a risk factor of DVT in patients undergoing spinal interbody fusion surgery. We speculate that it is mainly because of the low back pain due to operative wound, since we did not routinely apply other analgesics to the patients after spine surgery, except patient controlled analgesia prescribed by an anesthetist. Fearful of pain, the patients after surgery did less lower limb exercise on bed, resulting in worse blood circulation of lower limbs, which may contribute to DVT development.

Taken all together, it may have detected several interesting correlations in this study that will help in understanding DVT development in patients with spinal interbody fusion surgery. As well, some findings have been identified as indicators of an early alert for the development of postoperative DVT. The Third Hospital of Hebei Medical University is the biggest orthopedic hospital in Hebei Province; hence, the biggest limitation of the study is the inevitable inclusion of relatively more acute and severe patients in this study. The merit of this study is notably the preoperative screening of VTE. Thus, some of patients with VTE before surgery were excluded, which has decreased 1 important confounder that may falsely increase postoperative DVT incidence.

In conclusion, in this cross-sectional study, advanced age, high VAS scores during the first 3 days after surgery, and large amount of blood transfusion were risk factors for lower limb DVT in patients undergoing spinal interbody fusion. On the contrary, HDL was more likely a protective factor, not a risk factor for postoperative DVT incidence. As well, the logistic regression model may contribute to an early evaluation postoperatively for the risk of lower limb DVT in patients undergoing lumbar interbody fusion surgery.

## References

[1] Bang SM Jang MJ Oh D Korean guidelines for the prevention of venous thromboembolism. *J Korean Med Sci* 2010; 25:1553–1559. 21060742 10.3346/jkms.2010.25.11.1553 PMC2966990

[2] Moser KM Fedullo PF LitteJohn JK Frequent asymptomatic pulmonary embolism in patients with deep venous thrombosis. *JAMA* 1994; 271:223–225. 8277550

[3] Huisman MV Buller HR ten CJW Unexpected high prevalence of silent pulmonary embolism in patients with deep venous thrombosis. *Chest* 1989; 95:498–502. 2920574 10.1378/chest.95.3.498

[4] Strom RG Frempong-Boadu AK Low-molecular-weight heparin prophylaxis 24 to 36 hours after degenerative spine surgery: risk of hemorrhage and venous thromboembolism. *Spine* 2013; 38:E1498–E1502. 23873245 10.1097/BRS.0b013e3182a4408d

[5] Anderson FA Jr Wheeler HB Goldberg RJ A population-based perspective of the hospital incidence and case-fatality rates of deep vein thrombosis and pulmonary embolism. The Worcester DVT Study. *Arch Intern Med* 1991; 151:933–938. 2025141

[6] Geerts WH Code KI Jay RM A prospective study of venous thromboembolism after major trauma. *N Engl J Med* 1994; 331:1601–1606. 7969340 10.1056/NEJM199412153312401

[7] Si WT Zhang HG Sun YB Correlation analysis on plasma D-dimer level with deep venous thrombosis after spinal surgery. *Zhongguo Gu Shang* 2014; 27:405–408. 25167672

[8] Matsumoto MH Rodrigues LC Batalini LG Influence of blood coagulability after spinal surgeries. *Acta Ortop Bras* 2014; 22:235–239.doi:101590/1413-78522014220500930. 25328429 10.1590/1413-78522014220500930 PMC4199638

[9] Yang SD Liu H Sun YP Prevalence and risk factors of deep vein thrombosis in patients after spine surgery: a retrospective case-cohort study. *Sci Rep* 2015; 5:11834 sre 11834 doi:10 1038/. 26135271 10.1038/srep11834 PMC4488742

[10] Lee HM Suk KS Moon SH Deep vein thrombosis after major spinal surgery: incidence in an East Asian population. *Spine* 2000; 25:1827–1830. 10888952 10.1097/00007632-200007150-00014

[11] Yoshioka K Kitajima I Kabata T Venous thromboembolism after spine surgery: changes of the fibrin monomer complex and D-dimer level during the perioperative period. *J Neurosurg Spine* 2010; 13:594–599.doi:103171/20105SPINE09883. 21039150 10.3171/2010.5.SPINE09883

[12] Yukizawa Y Inaba Y Watanabe S Association between venous thromboembolism and plasma levels of both soluble fibrin and plasminogen-activator inhibitor 1 in 170 patients undergoing total hip arthroplasty. *Acta Orthop* 2012; 83:14–21.doi:103109/174536742011652886. 22248164 10.3109/17453674.2011.652886 PMC3278651

[13] Yoshiiwa T Miyazaki M Takita C Analysis of measured D-dimer levels for detection of deep venous thrombosis and pulmonary embolism after spinal surgery. *J Spinal Disord Tech* 2011; 24:E35–E39.doi:101097/BSD0b013e3181f60603. 20975598 10.1097/BSD.0b013e3181f60603

[14] Dindo D Breitenstein S Hahnloser D Kinetics of D-dimer after general surgery. *Blood Coagul Fibrinolysis* 2009; 20:347–352.doi:10 1097/MBC 0b013e32832a5fe6. 19474701 10.1097/MBC.0b013e32832a5fe6

[15] Sudo A Wada H Nobori T Cut-off values of D-dimer and soluble fibrin for prediction of deep vein thrombosis after orthopaedic surgery. *Int J Hematol* 2009; 89:572–576.doi:101007/s12185-009-0323-4. 19430861 10.1007/s12185-009-0323-4

[16] Hamidi S Riazi M Cutoff values of plasma d-dimer level in patients with diagnosis of the venous thromboembolism after elective spinal surgery. *Asian Spine J* 2015; 9:232–238.doi:104184/asj201592232. 25901235 10.4184/asj.2015.9.2.232 PMC4404538

[17] Takahashi H Yokoyama Y Iida Y Incidence of venous thromboembolism after spine surgery. *J Orthop Sci* 2012; 17:114–117.doi:101007/s00776-011-0188-2. 22222443 10.1007/s00776-011-0188-2

[18] Buerba RA Giles E Webb ML Increased risk of complications after anterior cervical discectomy and fusion in the elderly: an analysis of 6253 patients in the American College of Surgeons National Surgical Quality Improvement Program database. *Spine* 2014; 39:2062–2069. 25271519 10.1097/BRS.0000000000000606

[19] Tominaga H Setoguchi T Tanabe F Risk factors for venous thromboembolism after spine surgery. *Medicine (Baltimore)* 2015; 94:e466 doi:101097/MD0000000000000466. 25654385 10.1097/MD.0000000000000466 PMC4602703

[20] Yoshioka K Murakami H Demura S Prevalence and risk factors for development of venous thromboembolism after degenerative spinal surgery. *Spine* 2015; 40:E301–306.doi:101097/BRS0000000000000727. 25494320 10.1097/BRS.0000000000000727

[21] McClendon J Jr Smith TR O'Shaughnessy BA Time to event analysis for the development of venous thromboembolism after spinal fusion >5 levels. *World Neurosurg* 2015; doi:101016/jwneu201503068. 10.1016/j.wneu.2015.03.068 25871780

[22] Platzer P Thalhammer G Jaindl M Thromboembolic complications after spinal surgery in trauma patients. *Acta Orthop* 2006; 77:755–760. 17068706 10.1080/17453670610012944

[23] Oda T Fuji T Kato Y Deep venous thrombosis after posterior spinal surgery. *Spine* 2000; 25:2962–2967. 11074685 10.1097/00007632-200011150-00019

[24] Koo KH Choi JS Ahn JH Comparison of clinical and physiological efficacies of different intermittent sequential pneumatic compression devices in preventing deep vein thrombosis: a prospective randomized study. *Clin Orthop Surg* 2014; 6:468–475. 25436073 10.4055/cios.2014.6.4.468 PMC4233228

[25] Jaffer AK An overview of venous thromboembolism: impact, risks, and issues in prophylaxis. *Cleve Clin J Med* 2008; 75 Suppl 3:S3–6. 18494222 10.3949/ccjm.75.suppl_3.s3

[26] Cox JB Weaver KJ Neal DW Decreased incidence of venous thromboembolism after spine surgery with early multimodal prophylaxis: clinical article. *J Neurosurg Spine* 2014; 21:677–684.doi:103171/20146SPINE13447. 25105337 10.3171/2014.6.SPINE13447

[27] Knudson MM Morabito D Paiement GD Use of low molecular weight heparin in preventing thromboembolism in trauma patients. *J Trauma* 1996; 41:446–459. 8810961 10.1097/00005373-199609000-00010

[28] Schizas C Neumayer F Kosmopoulos V Incidence and management of pulmonary embolism following spinal surgery occurring while under chemical thromboprophylaxis. *Eur Spine J* 2008; 17:970–974.doi:101007/s00586-008-0668-z. 18421483 10.1007/s00586-008-0668-z PMC2443263

[29] Brambilla S Ruosi C La Maida GA Prevention of venous thromboembolism in spinal surgery. *Eur Spine J* 2004; 13:1–8.doi:101007/s00586-003-0538-7. 14610663 10.1007/s00586-003-0538-7 PMC3468034

[30] Anderson FA Jr Spencer FA Risk factors for venous thromboembolism. *Circulation* 2003; 107:I9–16.doi:101161/01CIR000007846907362E6. 12814980 10.1161/01.CIR.0000078469.07362.E6

[31] Jameson SS James P Howcroft DW Venous thromboembolic events are rare after shoulder surgery: analysis of a national database. *J Shoulder Elbow Surg* 2011; 20:764–770.doi:101016/jjse201011034. 21420324 10.1016/j.jse.2010.11.034

[32] Epstein NE Intermittent pneumatic compression stocking prophylaxis against deep venous thrombosis in anterior cervical spinal surgery: a prospective efficacy study in 200 patients and literature review. *Spine* 2005; 30:2538–2543. 16284592 10.1097/01.brs.0000186318.80139.40

[33] Hohl JB Lee JY Rayappa SP Prevalence of venous thromboembolic events after elective major thoracolumbar degenerative spine surgery. *J Spinal Disord Tech* 2015; 28:E310–E315.doi:10.1097/BSD.0b013e31828b7d82. 23511649 10.1097/BSD.0b013e31828b7d82

[34] Kebaish KM Awad JN Spinal epidural hematoma causing acute cauda equina syndrome. *Neurosurg Focus* 2004; 16:e1. 15202871

[35] Lawton MT Porter RW Heiserman JE Surgical management of spinal epidural hematoma: relationship between surgical timing and neurological outcome. *J Neurosurg* 1995; 83:1–7.doi:103171/jns19958310001. 7782824 10.3171/jns.1995.83.1.0001

[36] Paiement GD Wessinger SJ Harris WH Cost-effectiveness of prophylaxis in total hip replacement. *Am J Surg* 1991; 161:519–524. 1903606 10.1016/0002-9610(91)91124-2

[37] Goldhaber SZ Risk factors for venous thromboembolism. *J Am Coll Cardiol* 2010; 56:1–7.doi:101016/jjacc201001057. 20620709 10.1016/j.jacc.2010.01.057

